# Isolation of *N*-Ethyl-2-pyrrolidinone-Substituted Flavanols from White Tea Using Centrifugal Countercurrent Chromatography Off-Line ESI-MS Profiling and Semi-Preparative Liquid Chromatography

**DOI:** 10.3390/molecules26237284

**Published:** 2021-11-30

**Authors:** Weidong Dai, Maria Ramos-Jerz, Dongchao Xie, Jiakun Peng, Peter Winterhalter, Gerold Jerz, Zhi Lin

**Affiliations:** 1 Key Laboratory of Tea Biology and Resources Utilization, Ministry of Agriculture, Tea Research Institute, Chinese Academy of Agricultural Sciences, 9 Meiling South Road, Hangzhou 310008, China; daiweidong@tricaas.com (W.D.); xiedc511@gmail.com (D.X.); pengjiakun@tricaas.com (J.P.); 2 Institute of Food Chemistry, Technische Universität Braunschweig, Schleinitzstrasse 20, 38106 Braunschweig, Germany; m.ramos@tu-braunschweig.de (M.R.-J.); p.winterhalter@tu-braunschweig.de (P.W.)

**Keywords:** white tea, *Camellia sinensis*, *N*-ethyl-2-pyrrolidinone-substituted flavanols, epimer isolation, centrifugal partition chromatography, biphasic solvent system evaluation, *off-line* ESI-MS/MS metabolite profiling, semi-preparative liquid chromatography, inhibition *α*-glucosidase

## Abstract

*N*-Ethyl-2-pyrrolidinone-substituted flavanols (EPSF) are marker compounds for long-term stored white teas. However, due to their low contents and diasteromeric configuration, EPSF compounds are challenging to isolate. In this study, two representative epimeric EPSF compounds, 5′′′*R*- and 5′′′*S*-epigallocatechin gallate-8-C *N*-ethyl-2-pyrrolidinone (*R*-EGCG-cThea and *S*-EGCG-cThea), were isolated from white tea using centrifugal partition chromatography (CPC). Two different biphasic solvent systems composed of 1. *N*-hexane-ethyl acetate-methanol-water (1:5:1:5, *v*/*v*/*v*/*v*) and 2. *N*-hexane-ethyl acetate-acetonitrile-water (0.7:3.0:1.3:5.0, *v*/*v*/*v*/*v*) were used for independent pre-fractionation experiments; 500 mg in each separation of white tea ethyl acetate partition were fractionated. The suitability of the two solvent systems was pre-evaluated by electrospray mass-spectrometry (ESI-MS/MS) analysis for metabolite distribution and compared to the results of the CPC experimental data using specific metabolite *partition ratio K_D_* values, *selectivity factors* α, and *resolution factors* *R_S_*. After size-exclusion and semi-preparative reversed-phase liquid chromatography, 6.4 mg of *R*-EGCG-cThea and 2.9 mg of *S*-EGCG-cThea were recovered with purities over 95%. Further bioactivity evaluation showed that *R*- and *S*-EGCG-cThea possessed in vitro inhibition effects on *α*-glucosidase with IC_50_ of 70.3 and 161.7 μM, respectively.

## 1. Introduction

Tea (*Camellia sinensis*, Theaceae) is one of the most consumed beverages in the world due to its health benefits and pleasant flavor [1,2]. In comparison to the more popular green and black teas, white tea (Chinese: *bái chá*) is a rare product, although it has become increasingly popular in recent years [3,4,5,6]. White tea is a slightly fermented tea, undergoing a prolonged withering and drying processes. White tea is classified into Silver Needle (also called Baihaoyinzhen), White Peony (also called Baimudan), Gong Mei, and Shou Mei according to the differences in the raw tea leaf materials used. In Fujian province of China, the main region producing white tea, long-term stored white tea is generally considered to have better health benefits and commands a much higher commercial price [5].

Some studies have revealed that chemical differences in white tea products are caused by storage time and the resulting conditions. For volatile compounds, Qi et al. demonstrated that 40 differential aroma compounds, including five alcohols, seven aldehydes, ten ketones, eight esters, seven alkanes, and three heterocyclic compounds were able to discriminate the chemical profiles of the rapidly aged, naturally aged, and fresh white tea products. Zhu et al. investigated the enantiomeric distributions of 23 volatile lactones and terpenoids in white teas and found that *S*-linalool content and *S*/*R*-dihydro-actinidiolide ratio significantly decreased and increased, respectively with the change of storage time, thus serving as potential markers for the reliable differentiation of white teas stored over different time periods [6]. For non-volatile compounds, Ning et al. found that the contents of flavanols and amino acids decreased with increasing storage duration, while gallic acid increased [7]. In our recent studies, 7 novel 8-C *N*-ethyl-2-pyrrolidinone-substituted flavanol (EPSF) compounds were found linearly formed from flavan-3-ols and the amino acid theanine during the storage and were validated as marker compounds for long-term storage of white tea [8]. Furthermore, the EPSF contents in white teas could also be applied for the prediction of the storage duration [9].

EPSF is a unique class of flavanol derivatives with a *N*-ethyl-2-pyrrolidinone moiety at the C-6 or/and C-8 position, and has become a research hotspot in the area of tea chemistry [10]. As the C-6 or C-8 carbon atom connecting with the *N*-ethyl-2-pyrrolidinone moiety is chiral, there are two configurations of EPSF compounds, the *R*- and ***S***-configuration. Besides long-term stored white teas [8,9], EPSF compounds were also discovered in fresh Baimudan white tea [11], stored raw Pu-erh tea [12], ancient cultivated tea of Xi-Gui [13], yellow tea [14], pu-erh tea [15], and the leaves of *Camellia sinensis* var. *pubilimba* [16]. Although EPSF compounds were newly discovered in teas only several years ago, a number of studies have demonstrated that EPSF compounds possess various biological activities, e.g., antioxidant activity [15], anti-inflammatory activity [17], protective effects on human microvascular endothelial cell injury induced by hydrogen peroxide [15], and cell senescence induced by high glucose concentrations [13]. Furthermore, inhibitory effects on advanced glycation end products (AGEs) [11] and acetylcholinesterase (AChE) [16] have also been reported.

Epigallocatechin-gallate (EGCG) is one of the most abundant polyphenolic compounds in teas and the precursor compound with regard to the formation of the EPSF compounds *R*-EGCG-cThea and *S*-EGCG-cThea [8]. The contents of EGCG, *R*- and *S*-EGCG-cThea were approximately 47.1, 0.8, and 0.4 mg/g in stored white teas, respectively [8,18]. Due to their low contents, the occurrence of epimeric compounds, and the interference of high-content EGCG, the isolation procedures of *R*- and *S*-EGCG-cThea from white teas are extremely challenging and require various processing steps, which limit further investigation into the health benefits of epimeric EPSF compounds.

All-liquid chromatography methods such as *countercurrent chromatography* (CCC) and *centrifugal partition chromatography* (CPC) have been widely used for the isolation of natural target compounds from complex plant and food samples [19,20,21,22]. CPC uses two non-miscible liquids as the stationary phase and mobile phase. The stationary phase is retained inside the CPC rotor column system due high velocity rotation and the induced centrifugal field, while the mobile phase is pumped through the stationary phase. The main advantage of CPC over HPLC is the absence of solid chromatographic phase materials, which allows it to avoid any irreversible adsorption of target compounds to the separation system [18]. In this study, CPC was implemented in order to take advantage of the complementary liquid–liquid separation characteristics compared to classical solid phase chromatography combined with *off-line* ESI-MS detection for specific metabolite detection and visualization of co-elution effects in the recovered semi-preparative CPC fractions [23,24,25]. The resulting fortified mixture of EPSF compounds was cleaned by size-exclusion chromatography and purified by semi-preparative HPLC to yield the pure epimers *R*-EGCG-cThea and *S*-EGCG-cThea. Further characteristic and principal tea polyphenols such as epigallocatechin (EGCG), gallocatechin-gallate (GCG) and epicatechin-gallate (ECG) were effectively fractionated in the CPC experiments.

## 2. Results and Discussion

### 2.1. LC-ESI-MS/MS Analysis of Polyphenols in Stored White Tea

**Figure 1:**
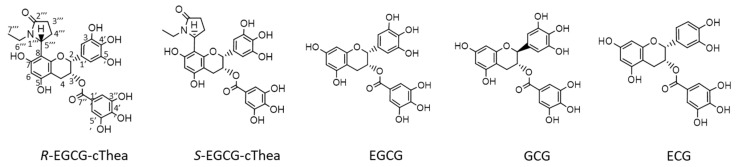
Chemical structures of *R*-EGCG-cThea, *S*-EGCG-cThea, epigallocatechin-gallate (EGCG), gallocatechin-gallate (GCG), and epicatechin-gallate (ECG).

**Figure 2:**
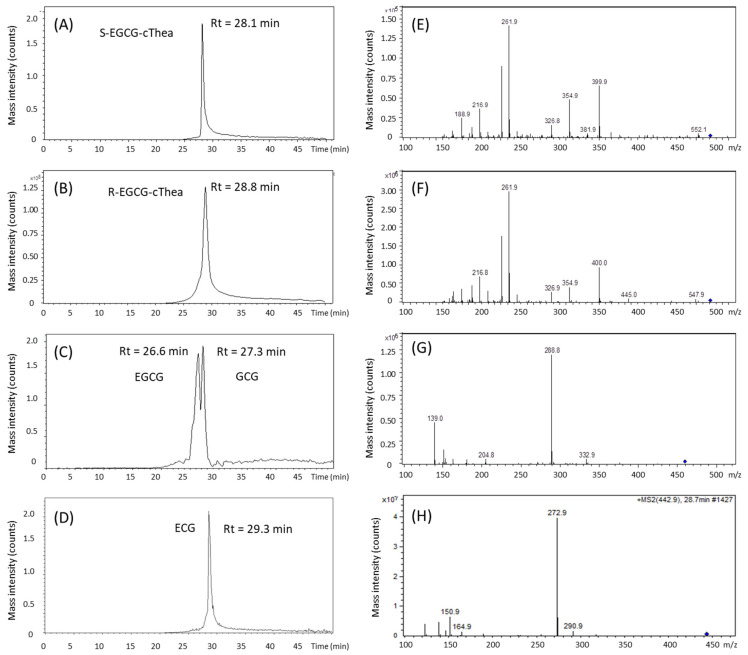
LC-ESI-MS selected ion chromatogram with [M + H]^+^ -signals of (**A**) *S*-EGCG-cThea (*m/z* 570), (**B**) *R*-EGCG-cThea (*m/z* 570), (**C**) EGCG and GCG (*m/z* 459), and (**D**) ECG, and ESI-MS^2^ fragment ions of (**E**) *S*-EGCG-cThea (*m/z* 570), (**F**) *R*-EGCG-cThea (*m/z* 570), (**G**) EGCG and GCG (*m/z* 459), and (**H**) ECG (*m/z* 443).

In the isolation study, the two target compounds, *R*-EGCG-cThea and *S*-EGCG-cThea (cf. Figure 1) were evaluated for optimization of suitable CPC two-phase solvent systems (cf. Section 2.2) for semi-preparative pre-fractionation. For removal of the existing predominant flavanols EGCG, GCG and ECG, the analytical investigation of the principal contents and concentrations was required. In the first step, a liquid chromatography-mass spectrometry (LC-ESI-MS) method was established to quantify the amounts of EGCG, GCG, ECG, *R*-EGCG-cThea and *S*-EGCG-cThea. As displayed in Figure 2, the ESI-mass-analysis of the two stereoisomers *R*-, and *S*-EGCG-cThea ([M + H]^+^, *m/z* 570) generated identical MS/MS fragment ion profiles, only showing a few intensity differences. The retention time analysis, using a C_18_-HPLC column, revealed that *R*-EGCG-cThea (t_R_: 28.8 min) had a slightly longer retention time than the respective epimer, *S*-EGCG-cThea (t_R_: 28.1 min), as seen by injection of authentic reference material. Flavanols EGCG ([M + H]^+^, *m/z* 459), GCG ([M + H]^+^, *m/z* 459), and ECG ([M + H]^+^, *m/z* 443) were detected at 26.6, 27.3, and 29.3 min, respectively (Figure 2).

### 2.2. Evaluation and Prediction of Compound Specific Partition Ratio (K) Values by LC-ESI-MS Analysis of White Tea Extract

Because of the close HPLC retention times of EGCG, GCG, ECG, *R*-EGCG-cThea and *S*-EGCG-cThea (cf. Figure 2), we speculated that it is difficult to directly separate low-content *R*-EGCG-cThea and *S*-EGCG-cThea from high-content EGCG, GCG, and ECG using preparative C_18_-HPLC. A two-step separation method was thus used; the CPC was firstly applied to isolate and concentrate low-content EPSF compounds from white tea, then semi-preparative LC was used for epimeric separation of *R*-EGCG-cThea and *S*-EGCG-cThea.

Two suitable semi-polar solvent system families, *N*-hexane-ethyl acetate-methanol-water (HEMWat) with solvent compositions of 1:5:1:5, 1:4:1:4, 1.5:3.5:1.5:3.5, and 2:3:2:3 (*v*/*v*/*v*/*v*), and *N*-hexane-ethyl acetate-acetonitrile-water (HEAWat) with 0.7:3:1.3:5, 1:4:1:4, 1:3:1:5, and 1:5:1:5 (*v*/*v*/*v*/*v*) were pre-evaluated by thin-layer chromatography for the CPC separation of the *R*- and S-EGCG-cThea mixture for semi-preparative fractionation from other major concentrated polyphenolic compounds in white tea. The final selection was made on basis of specific target compound *partition ratio* (*K_D_*) values determined by LC-ESI-MS analysis of the respective phase layers from shake flask experiments with the crude white tea ethyl acetate solvent partition. The calculation of the LC-ESI-MS *peak area values* (A) of the target molecules in the phase layers led to compound specific *K_D_*-values, and predicted two suitable systems (cf. prediction data in Table 1, Supplementary Material, Figure S1a,b); HEMWat (1:5:1:5, *v*/*v*/*v*/*v*) and HEAWat (0.7:3:1.3:5, *v*/*v*/*v*/*v*) were thus selected for the CPC pre-fractionation of the *R*- and *S*-EGCG-cThea mixture. As the CPC separation was in the so-called *descending-mode* using the more organic phase as stationary phase, the following *equation* (peak area A *upper phase*/ peak area A *lower phase*) was used for the prediction of metabolite specific *K_D_*-values (cf. Supplementary Material, Figure S1a,b). The polyphenol crude partition dissolved in the two-phase solvent systems HEMWat (experiment: CPC-1) and HEAWat (CPC-2) resulted in rather low *K_D_*-ratios for the target compound mixture *R*-/S-EGCG-cThea, with values of 0.41 and 0.64, respectively (cf. Table 1, Supplementary Material, Figure S1a,b). The predicted *K_D_*-values for the classical tea polyphenols such as EGCG and ECG ranged at much higher *K_D_*-values (0.8 to 4.8) (cf. Table 1) and suggested a successful clean-up process for the CPC separation stage. The two selected systems also met the empirical rule that suitable *K_D_*-values of target compounds in liquid–liquid countercurrent chromatography should be in the range of 0.5 ≤ *K_D_* ≤ 1.0 [26]. As the *elution-extrusion* methodology of *Berthod* et al. [27] was applied, larger metabolite *K_D_*-values were also suitable for recovery of compounds in the *extrusion*-stage of the CPC operation.

### 2.3. Isolation of R-EGCG-cThea and S-EGCG-cThea from White Tea

#### 2.3.1. Extraction and Liquid/Liquid Partitioning

The white tea leaves were crushed and macerated with a solvent mixture of methanol and water (70:30, *v*/*v*). The methanolic extract was diluted with water at a ratio of 1:2 (*v*/*v*), then liquid–liquid partitioned between *N*-hexane, dichloromethane and ethyl acetate, respectively. Thin-layer chromatography and LC-ESI-MS analysis of the *N*-hexane, dichloromethane, ethyl acetate, and water phase showed that *R*-EGCG-cThea and *S*-EGCG-cThea were fortified and mainly extracted to the ethyl acetate phase.

#### 2.3.2. Semi-Preparative CPC Fractionation and HPLC-Purification of R- and S-EGCG-cThea

**Figure 3:**
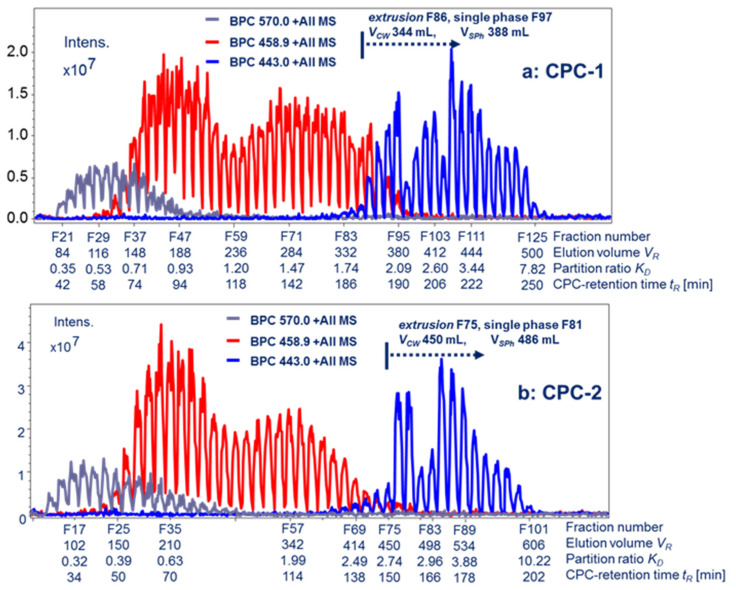
Metabolite visualization of semi-preparative CPC fractionations (CPC-1 and CPC-2) of white tea extract by *off-line* ESI-MS/MS analysis of fractions in the sequence of recovery, visualizing co-elution effects by use of selected single ion traces (ESI-MS positive mode, scan range *m/z* 100–2000). Detected target mass values for *R-/S*-EGCG-cThea (*m/z* 570), ECGC and GCG (*m/z* 459; EGCG eluted earlier than GCG), and ECG (*m/z* 443) as [M + H]^+^ signals. (**a**) *CPC-1* experiment (inj.: 500 mg) operated in the *elution/ extrusion*-mode (flow: 2.0 mL/min) with HEMWat (1:5:1:5, *v*/*v*/*v*/*v*), *extrusion*-mode at *switch volume V_CW_* (344 mL), velocity 1000 rpm, *corrected stationary phase retention S_F_* 89.5%, *corrected stationary phase V_S_* 179 mL (calculations cf. Supplementary Material, Data/Equations). (**b**) CPC-2 experiment (inj.: 500 mg) operated in the *elution/extrusion-mode* (flow: 3.0 mL/min) with HEAWat (0.7:3:1.3:5, *v*/*v*/*v*/*v*), *extrusion-mode* at *switch volume V_CW_* (450 mL), velocity 1000 rpm, *corrected stationary phase retention S_F_* 76.0%, *corrected stationary phase V_S_* 144 mL (calculations cf. Supplementary Material).

Two semi-preparative CPC separations were performed, each using 500 mg of white tea ethyl acetate partition but with two different solvent systems (HEMWat and HEAWat) (cf. Section 2.2), both being equivalent in polarity as evaluated by *K_D_*-values determined from the LC-ESI-MS analysis (cf. Section 2.2). The target CPC-fractions containing *R*- and *S*-EGCG-cThea were screened and identified by *off-line* ESI-MS injection profiling (cf. Section 3.5). As shown in Figure 3a,b, the mixture of *R*- and *S*-EGCG-cThea ([M + H]^+^ at *m/z* 570) eluted at much smaller retention volumes than EGCG ([M + H]^+^ *m/z* 459) and ECG (*m/z* 443). All fractions of CPC-1 and CPC-2 displaying the target ion [M + H]^+^ at *m/z* 570 were pooled. At this stage, the purity of the *N*-ethyl-2-pyrrolidinone-substituted EGCG compounds (*R*- and *S*-EGCG-cThea) reached 70–80% (LC-UV-ESI-MS analysis). Purification steps by size-exclusion chromatography on PVA 500 gel and semi-preparative C_18_-HPLC column using a previously established isocratic method [8,9] yielded pure epimers (6.4 mg *R*-EGCG-cThea and 2.9 mg *S*-EGCG-cThea) with purity > 95%.

### 2.4. Comparison of LC-ESI-MS Based Prediction Versus CPC Experimental K_D_-VALUES

In this CPC study for *R-/S*-EGCG-cThea recovery, metabolite specific *K_D_*-values in the biphasic solvent system predicted by LC-ESI-MS were compared to the real experimental CPC based *K_D_*-values (cf. Table 1, Supplementary Material, Figure S1a,b). This process of *K_D_*-comparison required the conversion of the CPC experimental times into a *partition ratio* (*K_D_*)-based chromatography scale, as published by Tran et al. [24] and *Grecco* et al. [25]. This approach is a unique way to compare the separation performance of equipment with different technical designs, such as CPC or CCC. The complete process of *K_D_*-calculation is illustrated in the section Supplementary Material/Equations by using the experimental CPC chromatography parameters, such as *retention volumes V_R_*, CPC rotor *column volume V_C_*, *stationary phase volume V_S_*, and the *stationary phase retention* values *S_F_* with the required formulas in consecutive steps. The determination of *K_D_* in the *extrusion*-mode requires a second formula using the so-called *switch-volume V_CW_* to calculate the results until infinity (cf. Supplementary Material/Equation (S7b)) [28]. The *off-line* injection profiles are using selected single ion traces, therefore a high precision for the detection of metabolites with their respective clearly detected *retention volumes V_R_* is guaranteed (cf. Table 1) which is the basis for the *K_D_*-calculation in the CPC experiments.

The comparison revealed (cf. Table 1) that the LC-ESI-MS predicted *K_D_*-values for most of the metabolites matched well to the CPC derived *K_D_*-mean $\bar{x}$ values (using the peak centers of CPC elution), and rather small off-sets for the calculation of Δ*K_D_* values occurred, although a large discrepancy was detected for the metabolite 443-ECG recovered from the *extrusion*-process in the CPC runs. During *extrusion* both phases are recovered, thus inconsistencies in the process are more likely to occur. It was very obvious that both CPC experiments displayed wide *retention volume* windows (*V_R_*), or Δ*K_D_*-ranges for metabolite elution (Table 1, Figure 3a,b). The addition of low concentrated acids to the solvent systems (such as the ion-pair forming reagent trifluoro acetic acid, TFA) might improve the CPC peak shapes, but could also lead to undesired artefact formation for recovered substances after vacuum-evaporation or lyophilization processing. Overall, both experiments with the HEMWat (CPC-1) and HEAWat (CPC-2) solvent system family displayed very similar *K_D_*-values in the prediction (cf. Table 1). The polarity of biphasic solvent system for all-liquid chromatography could be evaluated and compared using a method suggested by *Abbott* and *Kleiman* (1991) where Reichardt’s dye indicates by the wavelength of absorbance of the polarity attributes of the phase layers [28]. The visual results for the CPC-1 and CPC-2 solvent systems are displayed in Supplementary Material Figure S2, and indicate similar attributes of polarity.

### 2.5. Comparing Chromatographic Performance of CPC-1 and CPC-2

For the comparison of the chromatographic performance of CPC-1 and CPC-2 on white tea metabolites, the specific CPC mean $\bar{x}$ *K_D_*-values (Table 1) were used to calculate *separation factors α* and *resolution factors R_S_*. The procedure of calculations is displayed in Supplementary Material/Equations, and results for α− and *R_S_*-values are given in Table 2.

Both CPC separations resulted in rather small *α*-values for the metabolite pair *570 R*-/*S*-EGCG-cThea–459-EGCG (cf. Table 2, Figure 3a,b) (CPC-1: *α* 1.34 and CPC-2: *α* 1.11) with partial co-elution. These values indicated the insufficient separation of the target molecule mixture from 459-EGCG. A value of *α* > 1.5 would indicate a base-line separation. Nevertheless, the results from the *off-line* MS profile in both CPC runs proved that the largest amounts of principal polyphenols were clearly separated.

Unfortunately, the LC-ESI-MS analysis conducted on some of the respective target fractions of *570 R/S-EGCG-cThea* gave no indication of pre-fractionation of *R*- and *S*-EPSF-epimers by the CPC-steps using either solvent system.

The pair *570 R*-/*S*-EGCG-cThea-459-GCG was completely base-line separated, as seen by the respective *α*-values (CPC-1: 2.65; CPC-2: 2.50) (Table 2).

The isobar pair 459-EGCG-459-GCG is partly fractionated, as the peak widths and co-elution areas could be solely estimated by interpolation of the Gaussian peak-shapes. Further LC-ESI-MS analysis with respective retention times could clarify the exact elution windows or peak widths of the diastereomers in CPC.

The compound 443-ECG was fully separated from all existent polyphenolic compounds, as seen by largest *α*-values and recovery from CPC in the *extrusion*-mode.

A certain contradiction is seen in the results for CPC-2 as a better fractionation of the pair 459-GCG-443-ECG was achieved than for CPC-1 (cf. Figure 3a,b), although the *stationary phase retention S_F_* displayed solely 76.0%, compared to 84.5% (Table 2). The reason for this could be that the *extrusion* mode was started at a larger retention volume in CPC-2, and therefore better fractionation on the CPC rotor column might have occurred due to an extended time of *elution*.

The *resolution factor values R_S_* for CPC-1 and CPC-2 were calculated (Table 2) for the mentioned polyphenolic pairs, but were not further used for the discussion of chromatographic performance. The extremely large peak widths for *elution/extrusion* in CPC-1 and CPC-2 resulted in unacceptably low *R_S_*-values, as the peak broadness is a parameter in the *equation* (cf. Supplementary Material/Equation (S9)) [29]. The *separation factor α* solely uses the mean *K_D_*-value, and neglects the real peak width, as only the centers are used for calculations [29]. Nevertheless, it could be stated that α-values provide a much better assessment of the performance of broadened peak widths such as in these CPC applications. Overall, the visual inspection of separation by selected single ion traces (Figure 3a,b) clearly revealed that CPC-1 and CPC-2 provided a very efficient fractionation in the case of white tea polyphenols.

### 2.6. Inhibition Effect of R-EGCG-cThea and S-EGCG-cThea on α-Glucosidase

**Figure 4:**
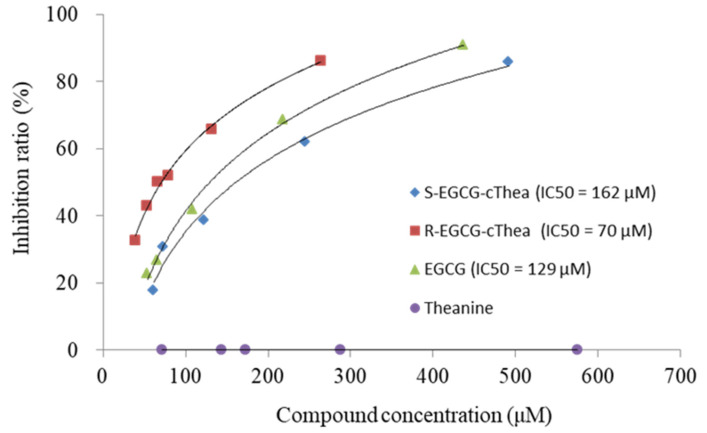
Dose-response inhibition curves and IC_50_ of EGCG, theanine, *S*-EGCG-cThea, and *R*-EGCG-cThea against α-glucosidase.

White tea was reported to have potential anti-hyperglycemic effects. For example, Xu et al. found that white teas have potent inhibitory in vitro effects on *α*-glucosidase and *α*-amylase activity, which are key enzymes related to type II diabetes [30]. The administration of white tea ethanolic extract for 14 days showed a significant effect on reducing fasting glucose level in streptozotocin-nicotinamide induced diabetic rats [31]. In addition, EGCG was reported to have potent inhibitory activity on *α*-glucosidase, with low IC_50_ concentration [32]. In this study, the inhibition effects of the isolated compounds of *R*-EGCG-cThea and *S*-EGCG-cThea, as well as their precursor compounds (EGCG and theanine) on *α*-glucosidase, were evaluated (Figure 4). The IC_50_ of EGCG was calculated to be 128.8 μM (Figure 4). Theanine showed no inhibitory effect on *α*-glucosidase. The IC_50_ of the epimer compounds *R*- and *S*-EGCG-cThea were found to be 70.3 and 161.7 μM, respectively (Figure 4). These results indicated differences that the inhibitory effect of EGCG on *α*-glucosidase was retained after *N*-ethyl-2-pyrrolidinone substitution at the C-8 position. However, the *R*-configuration *R*-EGCG-cThea displayed a higher *α*-glucosidase inhibitory activity than the *S*-form of *N*-ethyl-2-pyrrolidinone-substituted EGCG (*S*-EGCG-cThea). These results indicated differences in bioactivity between EPSF stereoisomers which are similar to the results obtained in our previous study, where *R*-EGCG-cThea showed slightly stronger anti-inflammatory property (i.e., suppressed the expression of NF-*κ*B-p65) compared to *S*-EGCG-cThea in lipopolysaccharide-stimulated macrophages [17].

## 3. Materials and Methods

### 3.1. Chemicals

Liquid chromatography grade methanol, ethyl acetate, acetonitrile, *N*-hexane, and dichloromethane were purchased from Merck (Darmstadt, Germany). EGCG (purity > 95.0%) and ECG (purity > 95.0%) was obtained from Sigma (St. Louis, MO, USA). Theanine (purity > 99.0%) was obtained from J&K Scientific, Ltd. (Beijing, China). *α*-Glucosidase 4-nitrophenyl-*β*-D-glucopyranoside (pNPG) was purchased from Megazyme (Wicklow, Ireland) and Yuanye (Shanghai, China), respectively. *R*-EGCG-cThea and *S*-EGCG-cThea had been previously synthesized and purified with purity > 95.0% in our laboratory [8,9].

### 3.2. Preparation of Sample Extract

125 g of a three-year stored white tea (stored in Fuding city, Fujian province, China, naturally preserved in a storehouse maintained at room temperature and 25−50% relative humidity) was ground into powder using a laboratory mill (M20, IKA, Germany) and extracted with 1875 mL methanol/water solution (70:30, *v*/*v*) at 70 °C for 1 h. After filtration with a paper filter at room temperature, the solution was distilled over vacuum to remove parts of methanol. Then, the extract solution was diluted with Nanopure^®^ water to the ratio 1:2 (*v*/*v*) for a liquid–liquid solvent partitioning procedure. Therefore, 200 mL *N*-hexane eight times and 200 mL dichloromethane eight times were used for defatting and removing semi-lipophilic metabolites. The resulting methanolic water phase was further extracted with 350 mL ethyl acetate ten times. After thin-layer chromatography analysis of the *N*-hexane, dichloromethane, ethyl acetate and water phase, the ethyl acetate phase partition was lyophilized, yielding 30.87 g of powder.

### 3.3. Evaluation of Partition Ratio (K_D_) Values Using LC-ESI-MS

The *partition ratio* (*K_D_*) values of the target compounds (*R*-EGCG-cThea and *S*-EGCG-cThea) were evaluated by LC-ESI-ion trap-MS/MS analysis (HCT Ultra ETD II, Bruker Daltonics, Bremen, Germany). Two suitable two-phase solvent systems composed of HEMWat (*N*-hexane-ethyl acetate-methanol-water) and HEAWat (*N*-hexane-ethyl acetate-acetonitrile-water) were tested in this study. Vials (20 mL) containing 1:1 mixture of the upper and lower phase (each 5 mL) of the above mentioned two-phase solvent systems were prepared. The vials were shaken vigorously with a vortex to achieve equilibration between upper and lower phases. After 10 min separation time of the phase layers, 1 mL of each phase was transferred separately into new vials and approximately 4 mg of the ethyl acetate soluble partition of white tea was added and mixed vigorously with a vortex. Aliquot volumes of the re-separated phase layers were injected to LC-ESI-MS for peak area analysis and prediction of target compound *K_D_*-values (cf. results Table 1). The *K_D_* value of each target compound was calculated as the mass intensity in areas in the stationary phase (upper phase) divided by the area value of the mobile phase (lower phase) (cf. Supplementary Material, Figure S1a,b, Equation (S1)).

### 3.4. CPC Isolation of the Mixture R- and S-EGCG-cThea from White Tea

The fast centrifugal partition chromatography apparatus (FCPC-Kromaton Technologies, Annony, France) was operated with a stainless semi-preparative rotor with mounted stainless steel separation disks (column volume *V_C_*: 200 mL). After gentle equilibration of the required solvents in a separatory funnel, the two phase layers were separated and briefly degassed before use in an ultrasonic bath. The prepared solvents were pumped with a preparative LC pump (solvent delivery system, K-501, Knauer Wissenschaftliche Geräte GmbH, Berlin, Germany). After complete filling of the stationary phase to the rotor column, the CPC system was started at 1000 rpm, while the mobile phase (lower phase) was pumped in *descending*-mode to reach the hydrodynamic equilibrium. In both CPC experiments, approximately 500 mg of CPC powder from liquid–liquid partitioning (cf. Section 3.2) was dissolved in 4.0 mL of each phase of the solvent system and filtered over a Chromafil Xtra GF-100/25 fiberglass membrane disc filter (1 μm pore size, 25 mm i.d., Macherey & Nagel, Düren, Germany), and then injected over a high-pressure injection system (Knauer Wissenschaftliche Geräte GmbH, Berlin, Germany) with a peek loop of 10 mL to the CPC system. The flow rates for CPC-1 and CPC-2 were 2.0 and 3.0 mL/min, respectively. After finishing the CPC separations in the so-called *descending elution*-mode (top-down direction in the rotor system) with the upper organic stationary phase and aqueous mobile phase, the biphasic solvent system which still contained metabolites in the column system was pushed out by pumping of the stationary phase at a lower spinning velocity (approx. 500 rpm) to the fraction collector. The recovery system was a SuperFrac (Pharmacia, Uppsala, Sweden), with type B racks collecting fractions during the *elution*- and *extrusion* process at every second minute. Aliquots of the in-sequence collected fractions of CPC-1- and CPC-2 were filled to HPLC vials (cf. Section 3.5) to perform the *off-line* metabolite elution profile by sequential ESI-MS/MS injections (cf. Figure 3a,b).

### 3.5. Off-Line ESI-MS/MS Injection Analysis for CPC Molecular Weight Metabolite Profiling

For a molecular weight guided detection of white tea metabolites from the semi-preparative CPC experiments CPC-1 and CPC-2, aliquot volumes of recovered fractions were *off-line* injected to an ESI-MS/MS-ion-trap mass-spectrometer (cf. system 3.3) in the sequence of the chromatographic *elution* and *extrusion* process. By selecting the full base-peak chromatogram trace (BPC) and selected single ion target traces, the semi-preparative results were projected as molecular weight-based chromatograms, which guided the accurate fractionation process. The acquired MS-data, including MS/MS fragmentation of seven precursor ions, were recorded in single data files. For sample preparation, aliquot volumes from CPC-fractions were filled to the HPLC vials. The *off-line* injections to the ESI-MS/MS were conducted by an independent and programmable autosampler system (AS-2000A, Merck-Hitachi, Tokyo, Japan). The injected fraction volumes (3 µL) were delivered to the ESI-MS device by an HPLC-pump (binary pump, G1312 A, 1100 Series, Agilent, Waldbronn, Germany) at a flow rate of 0.5 mL/ min, and the make-up solvent system used was composed of ACN/H_2_O (7:3, *v*/*v*). The time interval between the re-occurring injections was set to 2 min. Every observed injection peak corresponded to one injected vial from the CPC experiments. The selected ESI-MS traces (Figure 3a,b) displayed the mass spectrometry profile information of the compounds located in the respective CPC fraction(s).

The highest ion intensities in the generated base-peak chromatograms (BPC) at the scanning range of *m/z* 100–2000 were observed at 4.0 × 10^7^, presenting a good *signal-to-noise* ratio. The *N*-ethyl-2-pyrrolidinone-substituted EGCG compounds yielded a sensitive ion response in the positive ESI-ionization mode.

### 3.6. Further Isolation of Stereomeric R-EGCG-cThea and S-EGCG-cThea by Semi-Preparative Chromatography

The fractions of CPC-1 and CPC-2 containing the target compound mixture *R*- and *S*-EGCG-cThea (cf. Figure 3a,b) were pooled and further cleaned with a size-exclusion gravity column chromatography (PVA 500, Merck, Darmstadt, Germany). Then, the pure epimers *R*- and *S*-EGCG-cThea were isolated by using semi-preparative HPLC chromatography in the isocratic elution mode with acetonitrile/ water solution (50:50, *v*/*v*) as mobile phase using a reverse-phase C_18_ column (Zorbax RX-C18, 9.4 × 250 mm, 5 μm, Agilent, Palo Alto, CA, USA). Finally, 6.4 mg *R*-EGCG-cThea and 2.9 mg *S*-EGCG-cThea were obtained.

### 3.7. α-Glucosidase Inhibition Assay of R-EGCG-cThea and S-EGCG-cThea

The determination of α-glucosidase inhibitory activity of R-EGCG-cThea and S-EGCG-cThea compared to EGCG and theanine was carried out according to the method described by *Xu* et al. [30], with slight modifications. Compound solution (50 μL) was mixed with 0.1 M phosphate buffer (100 μL, pH 6.9 with 1 U/mL α-glucosidase). Then, the mixture was incubated in 96 well plates at 25 °C for 10 min. After incubation, 50 μL of 0.1 M phosphate buffer solution (pH 6.9 with 5 mM pNPG) was added into each well at timed intervals. Subsequently, the reaction mixture was incubated at 25 °C for 5 min. Before and after incubation, absorbance values were measured at λ 405 nm by a microplate reader (Infinite^®^ M200 Pro, TECAN, Männedorf, Switzerland). Buffer solution instead of compound solution was utilized as the control sample. The α-glucosidase inhibitory activity was presented as inhibition percentage, and was calculated as in the following equation: inhibition ratio (%) = (1 − As/Ac) × 100, where As and Ac are the absorbance values of the compound solution and of the control, respectively.

## 4. Conclusions

In the present study, centrifugal partition chromatography (CPC) and semi-preparative HPLC chromatography were used to isolate the low-concentrated epimeric EPSF compounds *R*-EGCG-cThea and *S*-EGCG-cThea from white tea extract. All-liquid chromatography provided complementary separation characteristics to solid phase chromatography, and the loss of target compounds by chemisorption was avoided [26]. Two semi-polar two-phase solvent systems from the HEMWat and HEAWat family were optimized to reach suitable *partition ratio K_D_* values and *separation factors* α between the EPSF target compounds and the residual principal tea polyphenolic compounds. The metabolite profiling by in-sequence *off-line* injection of CPC fractions to the ESI-MS detector traced with absolute precision the very minor concentrated *R-/S*-EGCG-cThea metabolites in the semi-preparative fractions. The CPC process with two different semi-polar solvent systems enabled recovery at low CPC retention volumes and clearly fractionated the other tea polyphenols, which displayed much larger *K_D_*-values. This two-dimensional chromatography approach (2D: CPC x C_18_ HPLC) delivered the pure diastereomers. As the retention times for C_18_-HPLC of the target metabolites appeared to be very similar (cf. Supplementary Material, Figures S1a and S2b), the 2D-approach appears to be the most effective method for large-scale isolation of these minor concentrated metabolites from white tea. A scale-up of the isolation protocol is feasible, as industrial sized CPC devices are available and the evaluated solvent systems could be easily transferred. An exemplary bioactivity evaluation showed that the in vitro inhibition IC_50_ of *R*- and *S*-EGCG-cThea on *α*-glucosidase were 70.3 and 161.7 μM, respectively.

## Figures and Tables

**Table 1:** Target compound specific *K_D_*-values: LC-ESI-MS predicted solvent system *K_D_*-values vs. experimental CPC values determined by *off-line* ESI-MS injection profiling.

		CPC-1	Corrected *S_F_*	84.5%		CPC-2	Corrected *S_F_*	76.0%		
Target Compounds ESI-MS (+Mode) *m/z*: [M + H]^+^	Solv. System 1 *K_D_*-Prediction (LC-MS) Cf. Details in Supplementary Material, Figure S1	*Exp.* Peak Range Fractions Retention Vol. ~[mL] Peak Width [mL]	*K_D_* Range Δ*K*_D_ Width W	CPC-1 Mean Value $\bar{x}$ *K_D_*	Δ*K_D_* = *pred. K_D_* − *exp. K_D_* CPC-1	Solv. System 2 *K_D_*-Prediction (LC-MS) Cf. Details in Supplementary Material, Figure S1	*Exp.* Peak Range Fractions Retention Vol. ~[mL] Peak Width [mL]	*K_D_* Range Δ*K_D_* Width W	CPC-2 Mean Value $\bar{x}$ *K_D_*	Δ*K_D_* = *pred. K_D_* − *exp. K_D_* CPC-2
***m/z* 570** ***570 R*-/*S*-EGCG-cThea**	0.41	F21–F47 84–188 104	0.35–0.93 0.58	0.64	−0.23	0.72	F13–F47 66–282 216	0.15–1.57 1.42	0.86	−0.14
***m/z* 459** **Epigallocatechin-Gallate ** **459-EGCG**	0.88	F29–F59 * 116–236 120	0.53–1.20 0.67	0.86	+0.02	0.95	F23–F47 * 138–282 144	0.35–1.57 1.22	0.96	−0.01
***m/z* 459** **Gallocatechin-Gallate ** **459-GCG**	1.57	F59–F95 * 236–380 144	1.20–2.20 1.00	1.70	−0.13	1.92	F47–F75 * 282–450 168	1.57–2.74 1.17	2.15	−0.23
***m/z* 443** **Epicatechin-Gallate** **443-ECG**	3.13	F83–F125 332–500 168	1.74–7.82 6.08	4.78	−1.65	3.06	F69–F101 414–606 192	2.49–10.22 7.73	6.35	−3.29

* The isobars 459-ECGC and 459-CGC were not base-line separated by CPC-1 and CPC-2, therefore the determined *K_D_*-ranges were estimated from the *off-line* ESI-MS profiling experiment.

**Table 2:** Comparison of values of *separation factor α* and *resolution factor R_S_* of metabolite pairs from semi-preparative CPC-1 and CPC-2.

Fractionated Compound Pairs	CPC-1 α-Value	CPC-2 α-Value	Δα [CPC-1 − CPC-2]	CPC-1 Resolution Factor R_S_	CPC-2 Resolution Factor R_S_	ΔR_S_ [CPC-1 − CPC-2]
**570 R-/S-EGCG-cThea/459-EGCG**	1.34	1.11	+0.23	0.35	0.08	+0.27
**570 R-/S-EGCG-cThea/459-GCG**	2.65	2.50	+0.15	1.34	1.00	+0.34
**570 R-/S-EGCG-cThea/443-ECG**	7.47	7.38	+0.09	1.24	1.20	+0.04
**459-EGCG/459-GCG**	1.98	2.24	−0.26	1.00	1.00	+0.02
**459-EGCG/443-ECG**	5.58	6.61	−1.03	1.17	1.20	−0.03
**459-GCG/443-ECG**	2.81	2.95	−0.14	0.87	0.94	−0.07

**Note:** * *Sufficient α*-values (values > 1.5) are underlined; Formulas for *α* = *K_D_*_2_/*K_D_*_1_ (whereas *K_D_*_2_ > *K_D_*_1_); Formula for *Rs* = 2 (*K_D_*_2_ − *K_D_*_1_)/(*W*_2_ + *W*_1_).

## Data Availability

The data that support the findings of this study are available from the corresponding authors on a reasonable request.

## Supplementary Materials

The following are available online. Figure S1. Selected single ion traces (ESI pos. mode) for target metabolites in the phase layers and calculated KD-values, Figure S2. Evaluation of the chosen biphasic solvent systems (CPC-1 and CPC-2) by use of Reichardt’s-dye, Equations (S1)–(S9).

## Sample Availability

Samples of the compounds are not available from the authors.
