# Supplementary material for: Isolation of N-Ethyl-2-pyrrolidinone-Substituted Flavanols from White Tea Using Centrifugal Countercurrent Chromatography Off-Line ESI-MS Profiling and Semi-Preparative Liquid Chromatography
Source: Molecules. 2021 Nov 30;26(23):7284. doi: 10.3390/molecules26237284 (PMC8658928; doi:10.3390/molecules26237284)
Supplement: Supplementary file 1 [file molecules-26-07284-s001.zip › molecules-1471060-supplementary.pdf]

Supplement Data

N-Ethyl-2-pyrrolidinone-substituted Flavanols from White Tea Using Centrifugal Countercurrent Chromatography Off-Line ESI-MS Profiling and Semi-preparative Liquid Chromatography

Weidong Dai [1,a], Maria Ramos-Jerz [2,a], Dongchao Xie [1], Jiakun Peng [1], Peter Winterhalter [2], Gerold Jerz [2,*] and Zhi Lin [1,*]

[1] Key Laboratory of Tea Biology and Resources Utilization, Ministry of Agriculture, Tea Research Institute, Chinese Academy of Agricultural Sciences, 9 Meiling South Road, Hangzhou, Zhejiang 310008, PR China; daiweidong@tricaas.com (W.D.); xiedc511@gmail.com (D. X.); pengjiakun@tricaas.com (J. P.); linzhi@caas.cn (Z. L.)

[2] Institute of Food Chemistry, Technische Universität Braunschweig, Schleinitzstrasse 20, Braunschweig 38106, Germany; m.ramos@tu-braunschweig.de (M. R.-J.); p.winterhalter@tu-braunschweig.de (P. W.); g.jerz@tu-braunschweig.de (G. J.)

* Correspondence: g.jerz@tu-braunschweig.de (G. J.); +49 531 391 7206 (G. J.); linzhi@caas.cn (Z. L.); +86-0571-86650617 (Z. L.)

[a] These authors contributed equally to this work.

Supplement Figure S1a/1b. *Prediction of suitable CPC solvent systems for the white tea sample by LC-ESI-MS analysis of target metabolite distributions in the phase layers of shake-flask experiments by calculation of specific metabolite partition ratio values* K_D.

Formula LC-ESI-MS prediction of partition ratio values : cf. results Table 1

K_D-value = peak area A upper phase / peak area A lower phase ***(Eq. 1)*** [Ito, 1996]

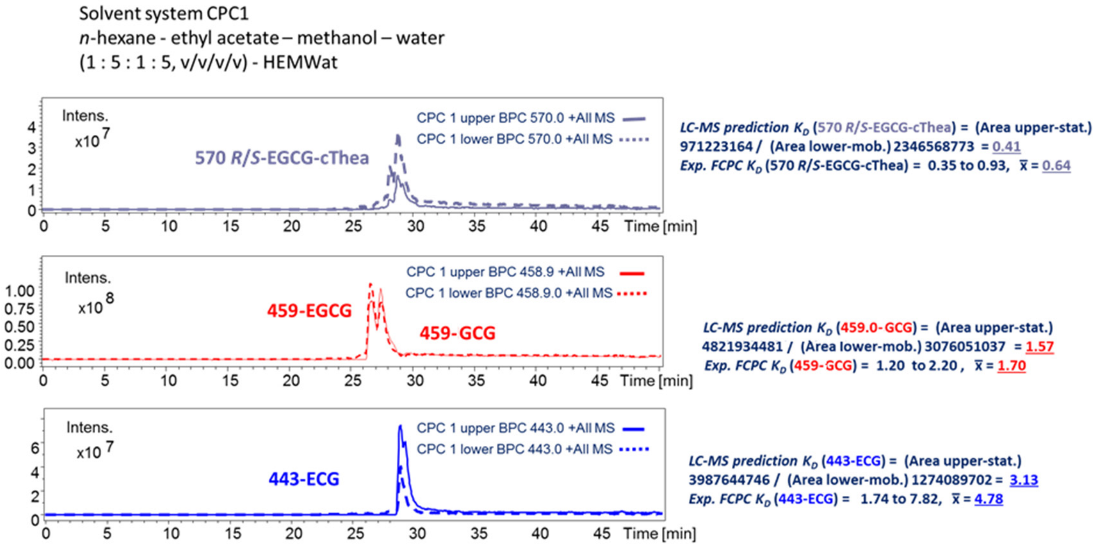

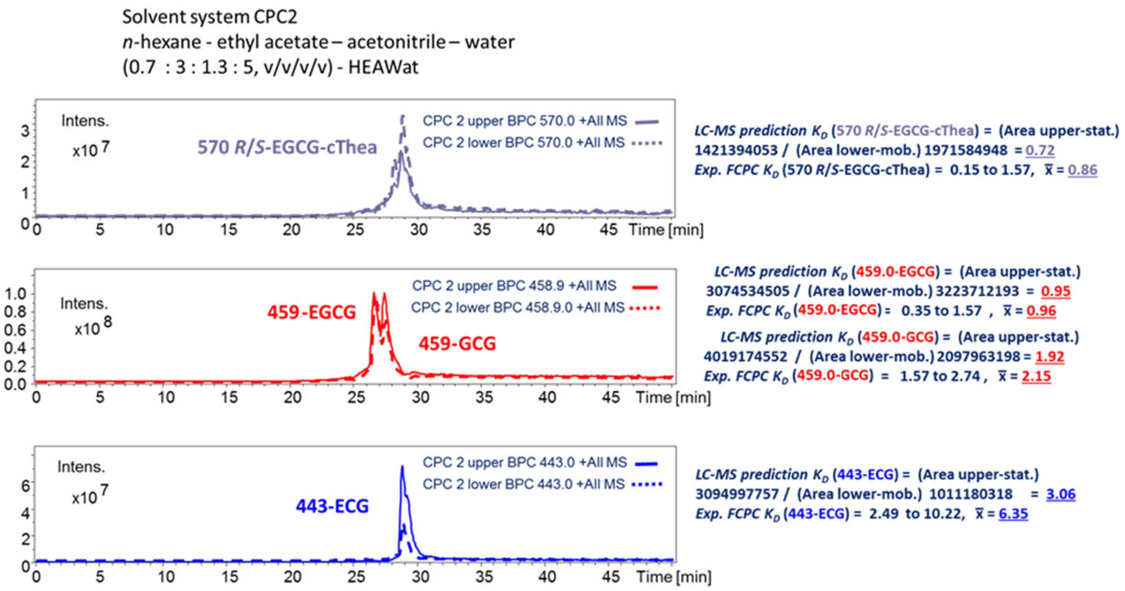

Suppl. Figure S1a/1b: Selected single ion traces (ESI pos. mode) for target metabolites in the phase layers and calculated K_D-values.

Supplement Figure S2. *Evaluation of the chosen biphasic solvent systems (CPC-1 and CPC-2) by use of Reichardt's-dye*

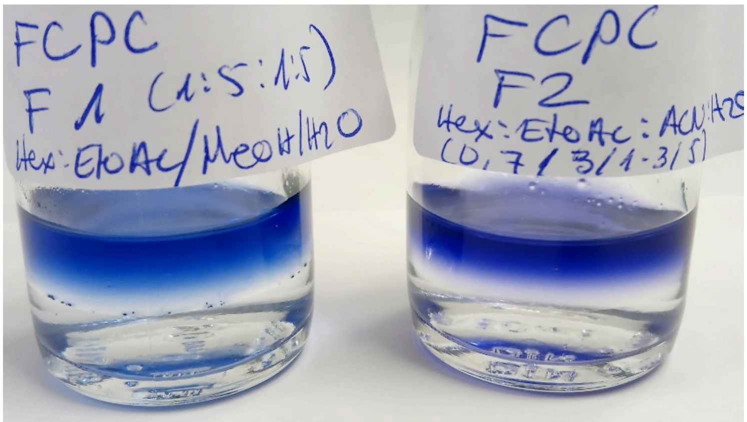

Reichardt's dye was suggested by Abbott & Kleiman (1991) for evaluation and classification of polarities of upper and lower phase layers in solvent systems being used for countercurrent chromatography (CCC) and therefore suitable in general for all-liquid separation techniques. Reichardt's dye absorbs light in the Vis-range (λ 400-900 nm) depending on solute polarities.

The two biphasic solvent systems were prepared in small volumes being used for CPC-1 (HEMWat 1 : 5 : 1 : 5, v/v/v/v) and CPC-2 (HEAWat 0.7 : 3 : 1.3 : 5, v/v/v/v). 1 mg of Reichardt's dye was added for each shake flask experiment. The wavelength absorbance in the blue range of the upper layers indicated a similar non-polar character for the two CPC solvent systems. The principal difference in the solvent system composition is the exchange of methanol against acetonitrile. This change of a solvent

component was compensated by the ratios adjusted to the metabolite distributions in the respective phase layers (as seen by similar K_D-value predictions in Suppl. Fig. S1).

Equations. *Calculation of CPC-chromatographic separation parameters*

In the performed CPC experiments, the chromatographic elution time was converted over *elution/retention volumes* V_R into their respective *partition ratio* values K_D (**cf. equations 2 - 7**). The K_D-based projection enables a better comparison between different liquid/liquid chromatography based machine designs such as *countercurrent chromatography* (CCC) [Grecco, 2019] and centrifugal partition chromatography (CPC). The experimental V_R values from the CPC runs for the tea metabolites were determined with high accuracy by the used *off-line* ESI-MS injection profiling of consecutive fractions as described below (also cf. 3.5).

Retention volume V_R = elution time [min] x flow rate [mL/ min] (**Eq. 2**)

The S_F **values** of the used solvent systems were determined by equation (**Eq. 3a-b**) using V_C (200 mL), and V_M resulting in the S_F value measured at the hydrodynamic equilibrium.
$V_S = (V_C - V_M)$ (**Eq. 3a**) $S_F = V_S / V_C \times 100\%$ (**Eq. 3b**)

CPC-1: S_F = 84.5%
CPC-2: S_F = 67.0%

V_S : retained experimental stationary phase volume
V_C : CPC column volume/capacity (200 mL)
V_M : volume of mobile phase take up to the coil at equilibrium of FCPC
V_{CM}: volume with change of elution to extrusion-mode (*switch volume*)
S_F : stationary phase retention
K_D: partition ratio

The determined S_F-value in the experiment is corrected by the *extra column volume* V_{Ext} (10 mL) of the connecting periphery tubing in the CPC set-up, using equations (**Eq. 4 - 6**)

Corrected $V_M = V_M - V_{ext}$ (**Eq. 4**)
CPC-1: *corr.* V_M = 31 mL - 10 mL = 21 mL
CPC-2: *corr.* V_M = 66 mL – 10 mL = 56 mL

Corrected $V_S = V_C$ - corrected V_M with CPC column volume V_C = 200 mL (**Eq. 5**)

CPC-1: *corr.* V_S = 200 mL - 21 mL = 179 mL
CPC-2: *corr.* V_S = 200 mL – 56 mL = 144 mL

It is important to note that a high S_F value directly correlate to a higher resolution and efficiency of the CPC-separation.

corrected S_F = corrected V_S / V_C (**Eq. 6**)
CPC-1: *corr.* S_F = 179 mL / 200 mL x 100% = 89.5%
CPC-2: *corr.* S_F = 144 mL / 200 mL x 100 % = 76.0% mL

The metabolite and solvent system specific partition ratio K_D values in the CPC run were calculated by the equations (**Eq. 7a, 7b**) (results cf. Suppl. Table 1). [Berthod et al.]

During e*lution-mode :* K_D = **(V_R - corrected V_M) / corrected V_S** (**Eq. 7a**) (cf. Fig. 3a, 3b)
In the *extrusion-mode* from begin of single phase recovery:
$K_D = V_{CM} / (V_{CM} + V_C - V_R)$ [Berthod et al.] (**Eq. 7b**) (cf. Fig. 3a, 3b)

The ***separation factor α*** and ***resolution factors Rs*** depend on the distances between peaks and the peak widths that will be compared. The calculation of both factors depend on the determined K_D values.

$\alpha = K_{D2} / K_{D1}$ **(with** $K_{D2} > K_{D1}$**)** (**Eq. 8**) (cf. Table 2)

To calculate the *resolution factor* (R_S) of the two peaks together with their respective K_D values, the measured peak widths at the baseline were used, as seen in the next formula:

$R_S = 2\,(K_{D2} - K_{D1}) \;/\; (W_2 + W_1)$ (**Eq. 9**) (cf. Table 2)

Wn: peak width at baseline

References

Abbott, T.P; Kleiman, R., Solvent selection guide for counter-current chromatography. *J. Chromatogr.* **1991**, 538, 109-118.

Berthod, A.; Friesen, J.B.; Inui, T.; Pauli, G.F. Elution-extrusion countercurrent chromatography: theory and concepts in metabolic analysis, *Anal. Chem.* **2007**, 79, 3371–3382.

Grecco, S.S.; Letsyo, E.; Tempone, A.G.; Lago, J. H. G.; Jerz, G. Electrospray mass-spectrometry guided target isolation of neolignans from *Nectandra leucantha* (Lauraceae) by high performance- and spiral-coil countercurrent chromatography. *J. Chromatogr. A* **2019**, 1608, 460422.

Ito, Y. Golden rules and pitfalls in selecting optimum conditions for high-speed counter-current chromatography. *J. Chromatogr. A* **2005**, 1065, 145-168.
